# Radiation Dose Optimization in Interventional Cardiology: A Teaching Hospital Experience

**DOI:** 10.1155/2018/6912841

**Published:** 2018-04-15

**Authors:** M. K. Badawy, T. Clark, D. Carrion, P. Deb, O. Farouque

**Affiliations:** ^1^Monash Imaging, Monash Health, Clayton, VIC 3168, Australia; ^2^School of Health and Biomedical Sciences, RMIT University, Bundoora, VIC 3083, Australia; ^3^Department of Radiology, Austin Health, Heidelberg, VIC 3084, Australia; ^4^Department of Medical Physics, Austin Health, Heidelberg, VIC 3084, Australia; ^5^Department of Cardiology, Austin Health, Heidelberg, VIC 3084, Australia; ^6^Department of Medicine, University of Melbourne, Melbourne, VIC 3010, Australia

## Abstract

Radiological interventions play an increasingly relevant role in cardiology. Due to the inherent risks of ionizing radiation, proper care must be taken with monitoring and optimizing the dose delivered in angiograms to pose as low risk as possible to staff and patients. Dose optimization is particularly pertinent in teaching hospitals, where longer procedure times are at times necessary to accommodate the teaching needs of junior staff, and thus impart a more significant radiation dose. This study aims to analyze the effects of different protocol settings in routine coronary angiograms, from the perspective of a large tertiary center implementing a rapid dose reduction program. Routine coronary angiograms were chosen to compare baseline levels of radiation, and the dose imparted before and after dose optimization techniques was measured. Such methods included lowering dose per pulse, fluoroscopic pulse rates, and cine acquisition frame rates. The results showed up to 63% reduction in radiation dose without adverse impact on clinical or teaching outcomes. A 10 fps/low and 5 pps/low setting was found to achieve maximum dose optimization, with the caveat that settings require incremental changes to accommodate for patient complexities.

## 1. Introduction

Interventional cardiology procedures have increasingly become vital to the current day practice of cardiology. However, as they heavily rely upon ionizing radiation, close attention needs to be paid to the radiation dose imparted to decrease the risk of side effects. Radiation dose optimization is, therefore, an essential consideration in the practice of interventional cardiology. However, such optimization techniques must also deliver adequate information to meet the clinical needs of cardiologists as well as not hinder the training needs of junior staff in teaching hospitals.

Radiation-induced side effects have been well documented in the literature and are divided into two categories: deterministic [1] and stochastic [2]. Deterministic side effects include radiation-induced skin burns and hair loss while stochastic side effects materialize over a longer time frame, as in the case of radiation-induced carcinogenesis. From a clinical vantage point, radiation side effects have ramifications for both the clinician and the patient: the former from prolonged occupational exposure to radiation, and the latter from the procedure itself. As such, it is crucial to optimize radiation dose imparted to as low as reasonably achievable.

A critical factor in addressing dose optimization is monitoring the large number of routine coronary angiograms that occur at tertiary teaching hospitals. The radiation dose imparted in teaching hospitals can surpass reasonable limits due to the longer procedural duration necessary to accommodate teaching needs of junior doctors. Consequently, radiation dose parameters must be optimized to ensure that although fluoroscopic times may be unchanged, the radiation dose be reduced to an acceptable level. These changes should not impact on both the clinical outcomes and the training needs at teaching hospitals. Similarly, the dose imparted should be comparable to published and local dose levels to ensure safe radiation practice.

Dose optimization techniques explored in the literature target pulse rate and frame rate, as well as determining optimal positioning. Kuon et al. were able to achieve reductions in mean DAP from 53.9 to 12.9 Gy·cm^2^ for routine coronary angiography/angioplasty during a focused, year long study by reducing cinegraphic frames/runs, fluoroscopy time, finding optimal positioning of blinds and filters, and favouring low radiation views for visualisation of the left anterior descending (LAD) and diagonal arteries [3]. Similarly, A four-step program implemented by Seiffert et al. showed a 54% reduction in DAP following a frame rate reduction, the use of fluoroscopy storage, and strict application of beam collimators [4]. McFadden et al. showed that a dose reduction of up to 49% could be achieved in a pediatric phantom study by lowering the cine frame rate from 30 fps to 15 fps with small decreases in image resolution [5].

The premise behind this paper originated in the clinical need to reduce radiation dose imparted following a review of routine coronary angiograms. This survey is conducted via a department audit which deemed the dose at this hospital higher than literature values. This paper will analyze the effects of varying optimization protocols on routine coronary angiograms, from the perspective of a large metropolitan tertiary center implementing dose optimization techniques.

## 2. Methods

### 2.1. Setting

This study was conducted in a cardiac catheterization laboratory of a large Victorian metropolitan teaching hospital. The staff consisted of experienced cardiologists and members of the Royal Australian College of Physicians (RACP) as well as fellows and registrars. The radiographers rotating through the time frame of the study were of varying seniority, from interns to radiography supervisors. The fluoroscopic X-ray unit used in this study was a Shimadzu Trinias F8 (flat panel detector technology) which had undergone all quality control tests subject to Australian Regulations in the state of Victoria. The on-board DAP meter was assessed for accuracy and was within 5% of an independently calibrated detector at the typical kVp range for cardiology procedures.

### 2.2. Study Design

Before the commencement of this study, an audit was conducted on 30 coronary angiograms. This audit was done as part of hospital quality assurance and was independent of this study itself. The results of the audit showed that the median DAP was found to be 75 Gy·cm^2^, a level which exceeded current literature, 14–63 Gy·cm^2^ [6, 7].

All patients undergoing diagnostic coronary angiography, with or without an additional left ventriculogram (LV), between March and September 2016 were included in this study. Patients who also underwent a graft study, right heart study, aortogram, IVUS (intravascular ultrasounds), or any intervention on top of the routine cardiac diagnostic angiograms were excluded.

There were five observational phases in this study, each phase lasting one month. Phase 1 was a tailored radiation awareness talk which detailed the current radiation dose levels at the facility plus targeted advice on how to reduce this dose. Suggestions included collimation, adjustment of patient positioning, and recommendation in lowering the fluoroscopic time. Phases 2–5 are a gradual decrease of the cine acquisition frame and dose rate and the fluoroscopic screening pulse and dose rate.  Table 1 describes the frame rates, pulse rates, and dose settings in each of the phases of the study.

The data from each phase of this study are compared. The primary outcome measure was dose area product and reference air kerma. The secondary outcome measure was fluoroscopic time.

### 2.3. Data Collection

Radiation dose data, total study time, the number of acquisitions, patient age, sex, height, and weight is collected through the procedural radiation dose structured report (RDSR). All radiation quantities are measured using the on-board DAP detector. Data collection was automated using scripts written in Python to extract the above information, and data are collected following the completion of each patient case. The information regarding left ventriculogram and access site is gathered from the procedure files of cardiac technologists. Data relating to each phase of the study are collected during the duration of that period.

### 2.4. Statistical Analysis

A one-way ANOVA is used to analyze the effect of BMI and age on the measured outcomes, while for the nonnormal variables of air kerma, DAP, and fluoroscopy time, a Kruskal–Wallis H test is used. When a significant difference is detected in the nonnormal data, a pairwise comparison using a Mann–Whitney *U* test with Bonferroni *p* value adjustment is conducted. All categorical data were compared using a chi-squared test. Statistical analysis is performed on R V3.3.2.

### 2.5. Image Quality

Image quality was quantitatively assessed using a CIRS Model 901 image quality phantom which allows compliance with the NEMA Standard—XR21 for cardiovascular fluoroscopic benchmarking. This phantom was used to determine the image quality using the settings in each phase of the study. The maximum thickness of 30 cm was used to determine the image quality for a simulated large patient, and the 20 cm configuration of the phantom was used to simulate average sized patients. The resolution and motion unsharpness were assessed using the inbuilt line pair bar pattern, iodine targets, air cylinders, aluminum cylinders, and the moving wire targets. A radiographer and a medical physicist conducted the assessment on image quality. Where there was no agreement on the scores, a third radiographer was asked to assess.

Clinical image quality is evaluated on patient images by experienced cardiology consultants through the duration of each phase. The assessment is undertaken during procedures and verbally communicated at multidisciplinary meetings which included a consultant cardiologist, a physicist, and a radiographer. The following questions were asked: Is there a notable difference in image quality? Is the image quality sufficient to complete the procedure? Is there a need to increase the dose in specific angulations or views?

## 3. Results

A total of 491 patients are included in the study. A Kruskal–Wallis H test was conducted to detect a statistically significant difference between phase and each of DAP, air kerma, and acquisition time. A post hoc Mann–Whitney *U* test with Bonferroni *p* value adjustment was used to detect the difference within phases. Results are summarised in Tables 2, 3, and 4.

A Kruskal–Wallis H test showed that there was a statistically significant difference in DAP values between the different phases, χ^2^ (2) = 106.66, *p* < 0.0001. There was also a statistically significant difference in air kerma between the various phases, χ^2^ (2) = 89.606, *p* < 0.0001. However, there was no statistically significant difference in fluoroscopy times between the different phases, χ^2^ (2) = 6.14, *p*=0.189.

Furthermore, a Mann–Whitney *U* test with Bonferroni *p* value adjustment revealed a statistically significant difference in DAP and air kerma between specific phases (*p* < 0.001, in all instances), as seen in Table 3. There was no statistically significant difference in fluoroscopy time between phases.

Quantitative image quality assessment showed no reduction in temporal image quality for an average-sized patient between the phases. There was a slight reduction in image contrast during fluoroscopic runs when dropping from 7.5 normal to all subsequent phases. The lower density iodine contrast groups were not seen at any lower settings. There was no reduction in subject contrast between the different cine acquisition settings. Image resolution showed a slight decrease during fluoroscopic runs at 5 pps/low and a similar reduction when moving from 15 fps to 10 fps during cine acquisitions. Similar reductions across the phases are exhibited in the 30 cm phantom, albeit with a lower initial baseline in image quality at the highest dose settings. The results are summarised in Tables 5 and 6.

Cardiologist feedback concerning the clinical image quality during every phase was that following the initial change: the first procedure would appear slightly grainy. The graininess was unnoticed following one or two procedures as the eye adjusted to the new settings. The image quality was sufficient to complete the examination, and there was no need to increase the dose except for steep angulations on obese patients.

## 4. Discussion

Due to the need to accommodate for the training of junior staff, teaching hospitals can be associated with longer procedure times which result in an increased radiation dose. Following the education talks in Phase 1 of this study, the median procedure time was 4.5 (3.4–6.4) mins with a DAP of 60.2 (43–84.6) Gy·cm^2^. At the conclusion of the study, the fluoroscopy time is 4.2 (3.2–6.4) min, and the DAP was 28 (17.6–35.2) Gy·cm^2^. At the time of writing, there were no established national diagnostic reference levels for coronary angiograms in Australia; however, a median DAP of 39 Gy·cm^2^ is reported by a multicentre study [7].

**Figure 1:**
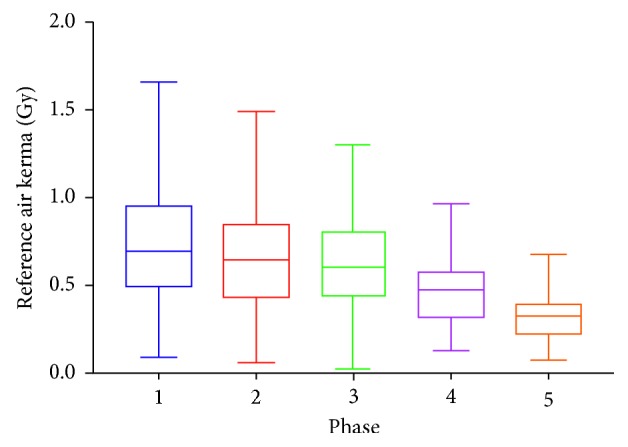
Median reference air kerma values during the different phases of the study. The error bars represent first and third quartile values.

**Figure 2:**
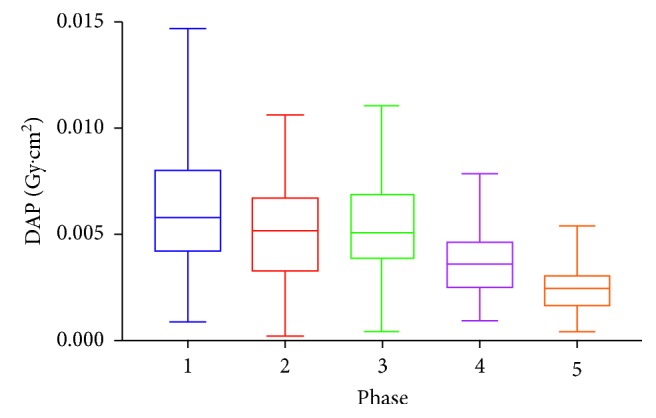
Median dose-area product values during the different phases of the study. The error bars represent first and third quartile values.

**Figure 3:**
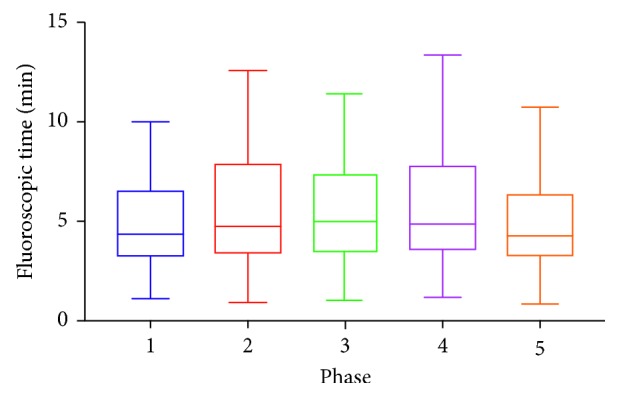
Median total fluoroscopic time values during the different phases of the study. The error bars represent first and third quartile values.

While a statistically significant dose reduction (DAP & Air Kerma) is not observed in every single phase, there is a downward trend in dose over the 5 phases (Figures 1 and 2). There is a statistically significant dose reduction observed when the frame rate is lowered from 15 to 10. The most prominent change to impact on the reduction is a change in “Dose per Pulse” rather than “Dose Rate.” For example, the most statistically significant reductions occurred when the dose setting is changed from normal to low on the fluoroscopic system. Comparing the DAP to the audit data found independently previous to this study, there has been a 63% reduction in radiation dose. No statistically significant change in total fluoroscopy time is noted (Figure 3), implying that procedure length did not need to be increased to account for reduced image clarity.

A short transition time is experienced in upskilling operators to use a lower image quality; however, not only is this quickly surpassed, but the clinical outcomes of the procedure are also not impacted. The incremental reduction in dose settings in this study allowed the operators to adapt to the decreased image quality. Feedback from consultant cardiologists is that the clinical image quality was adequate during every phase even though there was a slight increase in visible image noise. During routine procedures on average sized patients, no adjustments to dose rates were necessary throughout the examination. The only instance when an increase in dose settings was required is the combination of obese patients and steep angulations. These findings can be reflected quantitatively through the image quality phantom assessments. No reduction in temporal resolution was found; however, a slightly decreased image resolution and subject contrast was seen at the lower dose settings.

The presence of Phase 1, a targeted radiation awareness talk, is also deemed to be crucial to the success of the study, particularly given the different seniority of the staff base, as well as their rotating schedule. Regular review of essential concepts in dose optimization could play a fundamental role in reducing radiation dose. This is highlighted by the decrease in dose levels from 75 Gy·cm^2^ (prestudy) to 60 Gy·cm^2^ (phase 1) where the only difference between the two phases is the educational talk given before the commencement of phase 1.

Based on the findings of this study, we recommend that CA procedures be performed at 10 fps/low for cine acquisitions and 5 pps/low for fluoroscopic screening or equivalent low-dose pulse options in different fluoroscopic/angiographic systems as a standard. Patient characteristics or procedural complexities may necessitate changes to settings as instituted by the radiographer or operator; however, routine radiation safety talks can assist on how best to manage radiation dose settings to ensure that radiation dose to the patient remains as low as reasonably achievable. We believe using the incremental dose reduction methods employed in this study, and in conjunction with the equipment manufacturer, it may be possible to lower the frame and pulse rate even further to 7.5 fps and 3.75 pps.

## 5. Conclusion

A significant radiation dose reduction from lowering both frame rate and dose per pulse is seen in routine coronary cardiac angiograms. Furthermore, although a decreased image quality is observed, it did not impact on the diagnostic utility of the images, and no detrimental effects on the clinical outcomes or the teaching of the junior staff are reported back by senior cardiologists. The authors suggest that a 10 fps/low and 5 pps/low setting should be used as a standard in CA procedures to achieve dose optimization, with radiation settings being incrementally changed when necessary to account for patient complexities and characteristics.

## Figures and Tables

**Table 1:** Selected frame rate and pulse rate settings during the different phases of this study.

Phase	Frame rate (fps)/dose setting	Pulse rate (pps)/dose setting
1	15/normal	7.5/normal
2	15/normal	7.5/low
3	15/normal	5/low
4	10/normal	5/low
5	10/low	5/low

**Table 2:** Patient characteristics of phase-specific populations.

Characteristics	Phase 1	Phase 2	Phase 3	Phase 4	Phase 5	*p* value
*N*	129	79	156	76	51	
Male, no./total (%)	81/129 (63)	52/79 (66)	98/156 (63)	43/76 (57)	34/51 (67)	0.8
Age, mean (SD) (years)	64 (11)	64.1 (12.8)	65.2 (10.28)	65.9 (11.9)	63.4 (10.7)	0.6
Body mass index, mean (SD) (kg·m^−2^)	29.5 (5.65)	29.3 (6.2)	30.3 (6.6)	29.6 (6.2)	29.0 (4.5)	0.6

**Table 3:** The CA procedural characteristics of phase-specific populations.

Characteristics	Phase 1	Phase 2	Phase 3	Phase 4	Phase 5	*p* value
LV%	87	86	86	79	80	0.8
Radial %	57	72	66	61	59	0.2
No. of acquisitions	8.4 (1.6)	8.3 (1.7)	8.4 (1.5)	8.6 (1.5)	8.5 (1.7)	0.8
Air kerma (mGy)	0.68 (0.47–0.95)	0.63 (0.4–0.84)	0.59 (0.42–0.80)	0.46 (0.29–0.57)	0.3 (0.2–0.38)	<0.0001
DAP (Gy·cm^2^)	60.2 (43–84.6)	53.0 (36.5–69.7)	49.2 (36.5–68.4)	36.4 (25.2–46.9)	28.0 (17.6–35.2)	<0.0001
Total study time	4.5 (3.4–6.4)	4.8 (3.6–7.6)	5 (2.8–7.0)	5 (2.6–7.6)	4.2 (3.2–6.4)	0.2

**Table 4:** The phase-specific significant difference in cases where a significant difference is found (*p* < 0.001 in all instances where there was a statistically significant difference).

Phase	1	2	3	4	5
1	—	NSD	NSD	SD	SD
2	NSD	—	NSD	SD	SD
3	NSD	NSD	—	SD	SD
4	SD	SD	SD	—	SD
5	SD	SD	SD	SD	—

**Table 5:** Quantitative image quality results using the CIRS model 901 20 cm phantom for standard NEMA measurements.

		Phase 1		Phase 2		Phase 3		Phase 4		Phase 5	
		Fluoroscopy	Acquisitions	Fluoroscopy	Acquisitions	Fluoroscopy	Acquisitions	Fluoroscopy	Acquisitions	Fluoroscopy	Acquisitions
X-ray parameters	kVp	81	70	78	70	76	70	76	70	76	71
	Tube current (mA)	6.5	342	5	342	3	342	3	373	3	400
	Dose setting	Normal	Normal	Low	Normal	Low	Normal	Low	Normal	Low	Low
	Frame rate	7.5	15	7.5	15	5	15	5	10	5	10
	Added filteration	Al 2.0 + Cu 0.1	Al 1.0 + Au 0.01	Al 1.5 + Cu 0.6	Al 1.0 + Au 0.01	Al 1.5 + Cu 0.6	Al 1.0 + Au 0.01	Al 1.5 + Cu 0.6	Al 1.0 + Au 0.01	Al 1.5 + Cu 0.6	Al 2.0 + Cu 0.1

Static image quality	Line pairs	1.8	2.2	1.8	2.2	1.4	2.2	1.4	2	1.4	2
	Iodine group 1	6	8	6	8	6	8	6	6	6	8
	Iodine group 2	4	6	0	6	0	6	0	6	0	6
	Iodine group 3	0	0	0	0	0	0	0	0	0	0
	Iodine group 4	0	0	0	0	0	0	0	0	0	0
	Air cylinders	4	4	4	4	4	4	4	4	4	4
	Aluminum cylinders	4	4	4	4	4	4	4	3	4	3

X-ray parameters	kVp	75	71	77	71	77	71	77	72	77	73
	Tube current (mA)	5	432	5.7	432	3	432	3	400	3	399
	Dose setting	Normal	Normal	Low	Normal	Low	Normal	Low	Normal	Low	Low
	Frame rate	7.5	15	7.5	15	5	15	5	10	5	10
	Added filteration	Al 2.0 + Cu 0.1	Al 1.0 + Au 0.01	Al 1.5 + Cu 0.6	Al 1.0 + Au 0.01	Al 1.5 + Cu 0.6	Al 1.0 + Au 0.01	Al 1.5 + Cu 0.6	Al 1.0 + Au 0.01	Al 1.5 + Cu 0.6	Al 2.0 + Cu 0.1

Temporal image quality	Moving wires	3	4	3	4	3	4	3	4	3	4

**Table 6:** Quantitative image quality results using the CIRS model 901 30 cm phantom for standard NEMA measurements.

		Phase 1		Phase 2		Phase 3		Phase 4		Phase 5	
		Fluoroscopy	Acquisitions	Fluoroscopy	Acquisitions	Fluoroscopy	Acquisitions	Fluoroscopy	Acquisitions	Fluoroscopy	Acquisitions
X-ray parameters	kVp	118	90	113	90	110	90	110	102	110	102
	Tube current (mA)	11.4	746	11	746	7.1	746	7.1	341	7.1	340
	Dose setting	Normal	Normal	Low	Normal	Low	Normal	Low	Normal	Low	Low
	Frame rate	7.5	15	7.5	15	5	15	5	10	5	10
	Added filteration	Al 2.0 + Cu 0.1	Al 1.0 + Au 0.01	Al 1.5 + Cu 0.6	Al 1.0 + Au 0.01	Al 1.5 + Cu 0.6	Al 1.0 + Au 0.01	Al 1.5 + Cu 0.6	Al 1.0 + Au 0.01	Al 1.5 + Cu 0.6	Al 2.0 + Cu 0.1

Static image quality	Line pairs	1.6	1.8	1.4	1.8	1.2	1.8	1.2	1.6	1.2	1.6
	Iodine group 1	4	6	4	6	2	6	2	6	2	6
	Iodine group 2	0	0	0	0	0	0	0	0	0	0
	Iodine group 3	0	0	0	0	0	0	0	0	0	0
	Iodine group 4	0	0	0	0	0	0	0	0	0	0
	Air cylinders	4	4	4	4	4	4	4	4	4	4
	Aluminum cylinders	4	4	4	4	4	4	4	4	4	4

X-ray parameters	kVp	106	90	102	90	102	90	102	103	102	103
	Tube current (mA)	10	744	9	744	6.6	744	6.6	339	6.6	339
	Dose setting	Normal	Normal	Low	Normal	Low	Normal	Low	Normal	Low	Low
	Frame rate	7.5	15	7.5	15	5	15	5	10	5	10
	Added filteration	Al 2.0 + Cu 0.1	Al 1.0 + Au 0.01	Al 1.5 + Cu 0.6	Al 1.0 + Au 0.01	Al 1.5 + Cu 0.6	Al 1.0 + Au 0.01	Al 1.5 + Cu 0.6	Al 1.0 + Au 0.01	Al 1.5 + Cu 0.6	Al 2.0 + Cu 0.1

Temporal image quality	Moving wires	2	3	1	3	1	3	1	3	1	3
